# 

**Published:** 1860-03

**Authors:** 


### BUFFALO MEDICAL JOURNAL.

EDITED BY AUSTIN FLINT, Jr., M.D.,

Prof. of Physiology and Microscopic Anatomy in the New York Medical College.

And J. H. DOUGLAS, M.D.

NEW YORK:

CHARLES B. NORTON, APPLETON'S BUILDING,

346 AND 348 BROADWAY.

*LONDON*: H. BAILLIERE, 219 REGENT STREET. *PARIS*: J. B. BAILLIERE ET FILS, RUE HAUTEFEUILLE. *MADRID*: C. BAILLY-BAILLIERE, CALLE DEL PRINCIPE. *LEIPZIG*: BERNARD HERMANN, AGENT OF B. WESTERMANN & CO.

Hall, Clayton & Co., Printers 46 Pine St. N. Y.

Price $3 00 a Year, payable in Advance.

